# Age effects in Darwin’s finches: older males build more concealed nests in areas with more heterospecific singing neighbors

**DOI:** 10.1007/s10336-023-02093-5

**Published:** 2023-07-13

**Authors:** Antonia C. Huge, Nicolas M. Adreani, Diane Colombelli-Négrel, Çağlar Akçay, Lauren K. Common, Sonia Kleindorfer

**Affiliations:** 1 Konrad Lorenz Research Center for Behavior and Cognition, University of Vienna, Grünau im Almtal, 1030 Vienna, Austria; 2 Department of Behavioral and Cognitive Biology, University of Vienna, 1030 Vienna, Austria; 3 College of Science and Engineering, Flinders University, Adelaide, 5001 Australia; 4 Department of Psychology, Koç University, Istanbul, Turkey; 5 School of Life Sciences, Anglia Ruskin University, Cambridge, UK

**Keywords:** Neighbourhood, Acoustic landscape, Male age, Development, Habitat imprinting, Nest site

## Abstract

Nesting success tends to increase with age in birds, in part because older birds select more concealed nest sites based on experience and/or an assessment of prevailing predation risk. In general, greater plant diversity is associated with more biodiversity and more vegetation cover. Here, we ask if older Darwin’s finch males nest in areas with greater vegetation cover and if these nest sites also have greater avian species diversity assessed using song. We compared patterns in Darwin’s Small Tree Finch (*Camarhynchus parvulus*) and Darwin’s Small Ground Finch (*Geospiza fuliginosa*) as males build the nest in both systems. We measured vegetation cover, nesting height, and con- vs. heterospecific songs per minute at 55 nests (22 *C. parvulus*, 33 *G. fuliginosa*). As expected, in both species, older males built nests in areas with more vegetation cover and these nests had less predation. A novel finding is that nests of older males also had more heterospecific singing neighbors. Future research could test whether older males outcompete younger males for access to preferred nest sites that are more concealed and sustain a greater local biodiversity. The findings also raise questions about the ontogenetic and fitness consequences of different acoustical experiences for developing nestlings inside the nest.

**Supplementary Information:**

The online version contains supplementary material available at 10.1007/s10336-023-02093-5.

## Abstract (de)

**Alterseffekte bei Darwinfinken: Ältere Männchen bauen Nester mit mehr Vegetationsdecke und haben mehr heterospezifisch singende Nachbarn**.

Der Nesterfolg nimmt bei Vögeln tendenziell mit dem Alter zu, was zum Teil darauf zurückzuführen ist, dass ältere Vögel aufgrund ihrer Erfahrung und/oder der Einschätzung des vorherrschenden Prädationsrisikos besser versteckte Nistplätze auswählen. Im Allgemeinen ist eine größere Pflanzenvielfalt mit einer größeren Artenvielfalt und mehr Vegetationsdecke verbunden. Wir untersuchen hier, ob ältere Männchen der Darwinfinken in Gebieten mit größerer Vegetationsdecke nisten und ob diese Nistplätze auch eine größere Vogelartenvielfalt aufweisen, die anhand des Gesangs beurteilt wird. Wir verglichen die Muster beim Zwergdarwinfink (*Camarhynchus parvulus*) und Kleingrundfink (*Geospiza fuliginosa*), da die Männchen in beiden Systemen das Nest bauen. Wir haben an 55 Nestern (22 *C. parvulus*, 33 *G. fuliginosa*) die Vegetationsdeckung, die Nisthöhe und die kon- vs. heterospezifischen Gesänge gemessen. Wie erwartet, haben ältere Männchen beider Arten ihre Nester in Bereichen mit größerer Vegetationsdecke gebaut, und diese Nester wurden auch seltener ausgeraubt. Eine neue Erkenntnis ist, dass die Nester der älteren Männchen auch mehr heterospezifisch singende Nachbarn hatten. Künftige Untersuchungen könnten prüfen, ob ältere Männchen jüngere Männchen um den Zugang zu bevorzugten Nistplätzen verdrängen, die besser versteckt sind und eine größere lokale Artenvielfalt aufweisen. Die Ergebnisse werfen auch Fragen zu den Konsequenzen der unterschiedlichen akustischen Erfahrungen für die Entwicklung der Nestlinge im Nest auf.

## Introduction

In most bird species, breeding success and nesting behaviors change with age and experience, with experienced individuals usually better concealing their nests to improve nest survival (Marzluff 1988; Kleindorfer 2007a; Öst and Steele 2010; Horie and Takagi 2012). Females may prefer older males, particularly in species where males build the nest. For instance, in the Small tree finch (*Camarhynchus parvulus*), males build the nest and males increase the proportion of black plumage in the head and chin with each annual molt until they attain a completely black head in their fifth year (Kleindorfer 2007a). Female Darwin’s finches more quickly pair with older, darker males (Kleindorfer et al. 2019a), and pairs with an older male experienced higher breeding success because of lower nest predation (Kleindorfer 2007a). The proximate cause for lower nest predation in Small Tree Finches is thought to be nest placement, as nests of older males were more concealed and positioned higher up in the canopy, a pattern found in other studies too (Wappl et al. 2020; Heyer et al. 2021).

Males may use their own local breeding success as a patch quality cue that integrates the effect of various nest site attributes on breeding performance (Danchin et al. 1998; Doligez et al. 2002; Mariette and Griffith 2012). That is, males may return to a particular breeding site if they were previously successful at that site. Perhaps older males select safer nest sites based on experience and/or an assessment of prevailing predation risk. For example, Møller (1989) showed that older Northern Wheatear (*Oenanthe oenanthe*) males adjusted nesting height in relation to previous nesting outcome, with evidence that ground nesting birds may adjust their nest site and nest concealment according to predation risk. In general, older males have more breeding experience than younger males, though the mechanisms by which they evaluate previous experience and whether they make informed choices about future nest site selection is often unknown and can no doubt vary between systems.

In many species, individuals form breeding aggregations, which may carry significant benefits, such as protection from predators via a dilution effect that lowers individual detectability by predators, for example (Hamilton 1971; Rubenstein 1978). More individuals in an area can also increase predation risk by attracting predators to an area (Hassell et al. 1977; Hammond et al. 2007). While nesting in close proximity may increase the risk of extra-pair paternity or intra-specific brood parasitism (Brown and Brown 1989; Stewart et al. 2010), forming nesting associations with heterospecifics may circumvent that problem. For instance, in Darwin’s Finches on Santa Cruz Island, Galapagos, birds that nested in ‘mixed species associations’ with heterospecific neighbors had less nest predation (Kleindorfer et al. 2009). Close proximity to conspecifics also increases food competition, and local food abundance can affect the number and composition of individuals in an area (Forero et al. 2002; Booth 2004). Therefore, the neighborhood composition may influence nest survival, and in some systems, birds may avoid areas with many conspecific food competitors or favor areas with many heterospecific neighbors that provide additional anti-predator defense.

Nesting habitat also has consequences for the sensory experience of birds which in turn may influence their fitness. Songbirds are vocal production learners that acquire their song from conspecifics (Nelson et al. 1995; Catchpole and Slater 2008; Plamondon et al. 2008; Konishi 2010). Exposure to a tutor’s song that can become a song template is therefore a fundamental experience that guides song learning (Nottebohm 1972; Grant and Grant 1996). When individuals differ in song syllable composition and there is competition to transfer song syllable type to offspring (Evans and Kleindorfer 2016), fathers that nest in heterospecific neighborhoods may have an advantage to transmit their song type to offspring. Fathers in heterospecific neighborhoods should have less competition or interference for song syllable transmission compared to fathers with many conspecific neighbors because some offspring may attend to non-paternal conspecific song types (see also Katsis et al. 2018; 2023; Colombelli-Negrel et al. 2021). Learning and discrimination, including elementary forms of vocal production learning, can begin already during the egg in some songbird embryos. For example, Superb Fairywrens (*Malurus cyaneus*) produce a vocally acquired call after hatch copied from their (foster) mother’s in-nest call elements during incubation (Colombelli-Négrel et al. 2012), and embryos across avian taxa have been shown to learn to discriminate between sounds in ovo (Colombelli-Négrel and Kleindorfer 2017; Rivera et al 2018; Colombelli-Négrel et al. 2021). In an elegant field study, Mennill et al. (2018) showed that wild Savannah Sparrows (*Passerculus sandwichensis*) learned their songs from experimentally broadcast tutors placed near the nest in the wild. Thus, acoustic neighborhood is expected to play a significant role in vocal learning when vocal production learning embryos and nestlings are exposed to song in general, though to date, there are few studies that measure the acoustic neighborhood at the time of nesting across species.

There is a strong positive association between vegetation diversity and the avian diversity it supports (Lantz et al. 2011; Weisshaupt et al. 2011; La Sorte et al. 2020; Geladi et al. 2021). In forest systems, forests with more canopy cover and taller trees also sustained more bird species (Kirk and Hobson 2001). Similarly, in urban areas, avian species richness was higher in parks with more vegetation coverage (La Sorte et al. 2020). Vegetation cover may be associated with multi-level species richness as well as creating conditions for lower predation risk when songbirds select nest sites with more vegetation cover. In general, older males are expected to compete for and occupy better-quality territories (e.g., trees with broad canopy cover) that sustain more food resources (Sherry and Holmes 1989; Pärt 2001) and, when nest sites are more concealed in dense vegetation, lower predation risk (Hill 1988). A diverse heterospecific neighborhood could be a by-product of nest site preference for food or safety (associated with a dense vegetation cover), but in turn may facilitate other pathways, for example, acoustic habitat imprinting (see also Davis and Stamps 2004).

The aim of this study is to test if the nest sites of older male Small Ground Finches (*Geospiza fuliginosa*) and small tree finches differ in predictable ways from the nest sites of younger males, with specific attention to the singing activity of heterospecific and conspecific neighbors, as well as vegetation characteristics. First, we aimed to replicate the findings from Santa Cruz Island that older males occupy areas with more vegetation cover, in taller trees, and with higher nesting height in Floreana Island. Second, we test a new prediction that the acoustic neighborhood experienced by the offspring of older males will be more species rich with higher singing activity. Specifically, we predict that older males will nest in areas with more heterospecific neighbors and thus more heterospecific vocal activity while younger males will have more conspecific neighbors and more conspecific vocal activity. We also predict that the nest sites of older males will have more canopy cover and nests will be located higher up in taller trees. If nest predation is associated with singing activity (because sound alerts predators to an active area to search for nests), then we predict increased nest predation at nests with higher singing activity.

## Methods

### Study site and study species

This study was conducted on Floreana Island (− 1.299829, − 90.455674) during the onset of nesting and the Darwin’s finch breeding season that peaks during February and March and coincides with the onset of heavier rains usually during January and February (rainfall data can be accessed via https://www.galapagosvitalsigns.org). The nesting data were collected during February–March 2020 and February 2022 at 55 Darwin’s finch nests (Table 1), including Small Ground Finches (*G. fuliginosa*) (*N* = 33) and Small Tree Finches (*C. parvulus*) (*N* = 22). The nests were located across eight 100 × 200 m^2^ study plots in the highland *Scalesia* forest near Cerro Pajas or in two 100 × 200 m^2^ study plots at Asilo de la Paz, also a *Scalesia*-dominated forest.

**Table 1:** Sample size per nesting phase: the number of recordings and number of nests for each year

Nesting phase	Small ground finch				Small tree finch			
	Recordings		Nests		Recordings		Nests	
	2020	2022	2020	2022	2020	2022	2020	2022
Nest building	0	7	0	7	4	12	1	12
Incubation	26	0	10	0	4	1	3	1
Feeding	40	0	18	0	13	0	7	0
Number of nests	33				22			

From a long-term study using color-banded birds, Darwin’s finches are socially monogamous per brood (Grant and Weiner 1999; Keller et al. 2001; Kleindorfer 2007b). The onset of nesting occurs during the onset of heavier rains from January to March. Males use song and behavioral displays to defend small nesting territories (ca. 20 m^2^) against intruders. During higher rainfall years, the males may build several nests while singing to attract females, and eventually a female may choose one of the nests for egg-laying (Kleindorfer 2007a). However, during this study in 2020 and 2022, both years had low to moderately low rainfall and each male only built one display nest. The female is a uniparental incubator and the incubation phase lasts 12–14 days (Kleindorfer 2007a, b). Both parents provide food deliveries to nestlings until they fledge after 12–14 days (Kleindorfer et al. 2021a). Between 17 and 60% of highland Darwin’s finch nests are depredated across species and years (Kleindorfer 2007a, b; Kleindorfer and Dudaniec 2009; O’Connor et al. 2010; Cimadom et al. 2014; Kleindorfer et al. 2021b). In both species, males build a domed-shaped nest, often in *Scalesia pedunculata* trees. The avian vampire fly (*Philornis downsi*) is a major cause of nesting failure. On Floreana Island, newly built nests and nests with eggs do not contain *P. downsi*; only nests with chicks contain the avian vampire fly larvae (Common et al. 2019, 2023). In this study, 18 of the nests progressed to the chick stage in 2020 for which we also had information on number of *P. downsi* larvae and pupae at the time of nesting outcome; there was no association between male age and number of vampire flies (*r* = − 0.002, *p* = 0.992, *n* = 18). In 2022, a year with low rainfall, Darwin’s finches built a display nest, sang at the nest, but no eggs were laid and hence there were no avian vampire flies in finch nests in the 2022 data.

### Male age

**Fig. 1:**
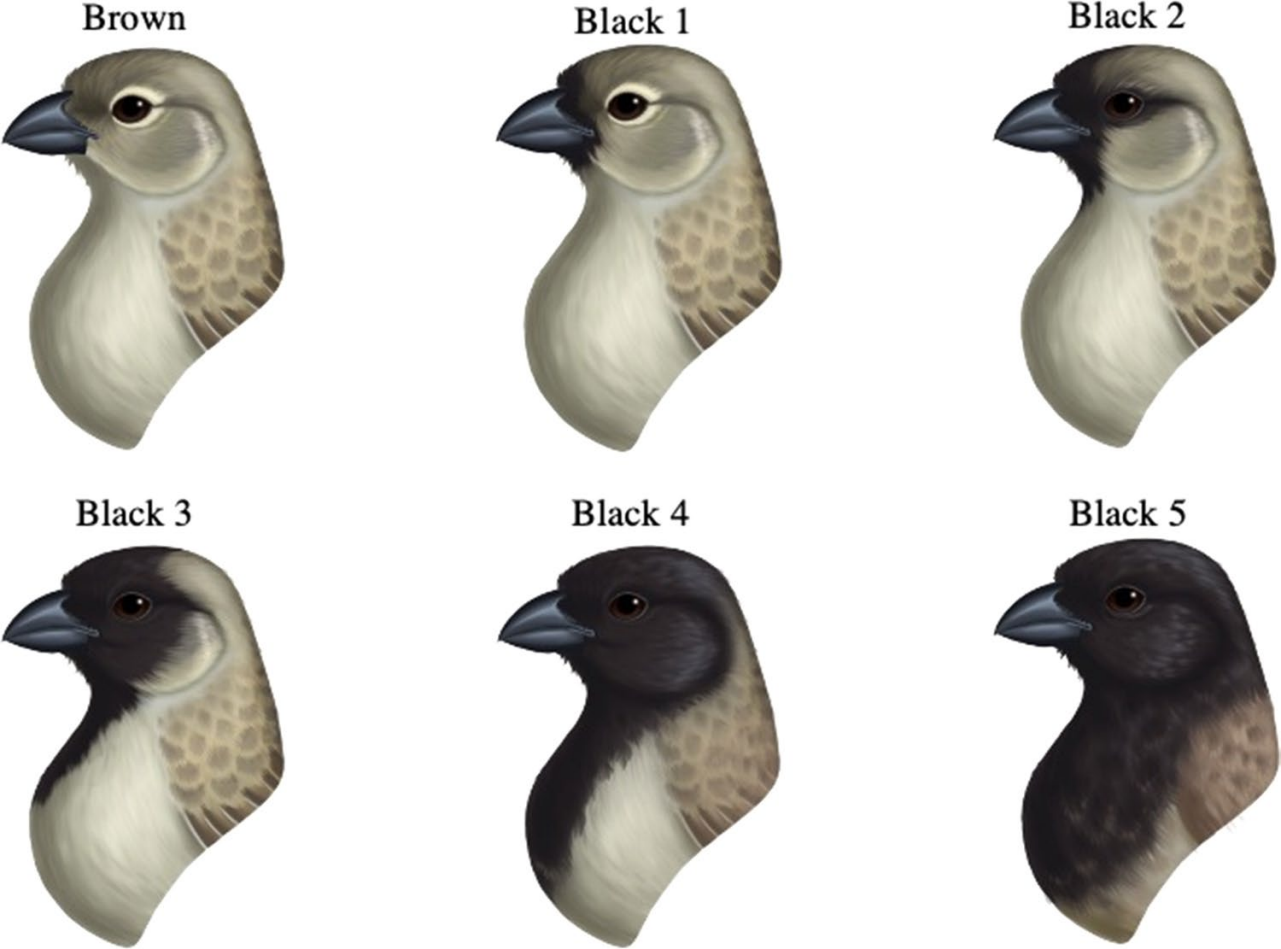
Changes in plumage coloration in male small tree finches with each annual molt. Males require, on average, 5 years to attain a fully black chin and crown. Brown to black 5 correspond to the color and age categories B0–B5 used, with B0 being yearling males and B5 including 5 years and older males. Copyright Lauren K. Common (colour figure online)

**Fig. 2:**
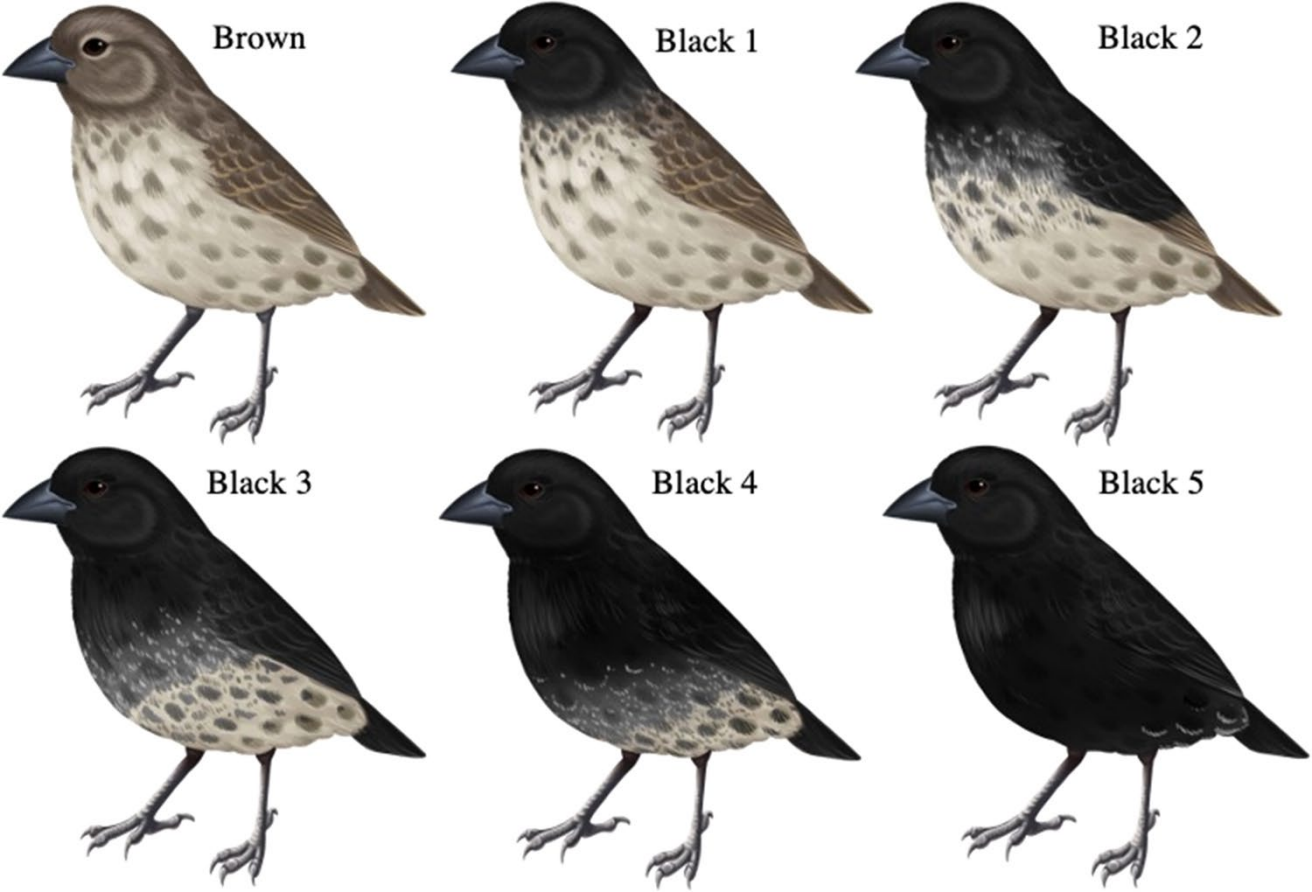
Changes in plumage coloration in male small ground finches with each annual molt. Males require, on average, 5 years to attain a fully black body. Brown to black 5 correspond to the color and age categories B0–B5 used, with B0 being yearling males and B5 including 5 years and older males. Copyright Lauren K. Common (colour figure online)

Darwin’s finch males can be aged in the field using binoculars based on the proportion of black plumage. In Darwin’s tree finches (Fig. 1), the proportion of black plumage on the chin and crown increases with each year of molt until they obtain a fully black head after about five years (Lack 1947; Kleindorfer 2007a; Langton and Kleindorfer 2019). In Darwin’s ground finches, the proportion of black plumage increases with each year of molt across five years (Fig. 2), until the male acquires full black plumage across its body (Grant and Grant 1987). Female Tree Finches remain olive green and female Ground Finches remain grayish across their lives and cannot be aged from plumage. The age classification of males is based on the six classes described by Grant and Grant (1987) for Small Ground Finches and by Kleindorfer (2007a) for small tree finches (Figs. 1, 2). The change in plumage with age gives us the rare opportunity to study the effects of age on nest site attributes, and how these are associated with the acoustic neighborhood near the nest, nest site vegetation, and predation outcome using an observational approach. The sample size per age class and species in this study is as follows: (i) small ground finches B1 = 2, B2 = 3, B3 = 4, B4 = 3, B5 = 21, and (ii) small tree finches B0 = 2, B1 = 1, B2 = 6, B3 = 2, B4 = 5, B5 = 6.

### Nest monitoring and nest site characteristics

Nests were monitored following our standardized protocol that we developed in 2000 and maintained throughout the study (Kleindorfer et al. 2014; Common et al. 2020). Nests were routinely inspected, with binoculars and ladder during 2004 to 2006, and since 2008 with a borescope, every three days during incubation and every two days during the nesting phase to confirm activity. Nesting height estimation was practiced using a laser pointer (LTI laser rangefinder) prior to field work, which we did using clearly visible trees on-campus at Flinders University, Australia. The laser rangefinder was first pointed at the base of the tree and then the top to compute two vertical angles, from which tree height was calculated. We calibrated among team members at the start of the field season and visually estimated tree height as meters above ground during field work.

We measured the following nest-site vegetation characteristics per nest within two weeks of nest building: (1) nesting height (m above the ground; ocular estimation after training with a laser pointer device on-campus at Flinders University), (2) nesting tree height (ocular estimation after training with a laser pointer device on-campus at Flinders University), (3) percentage canopy cover 1 m around the nest (ocular estimation after training calibration with botanist Heinke Jaeger in the field in 2020), and (4) percentage ground cover (ocular estimation calculated for 4 × 5 m quadrants at the base of the nest).

### Video and audio recordings at nests

Video and audio data were collected using GoPro cameras (GoPro Hero 7, GoPro Inc.) placed within 5 m of the nest. GoPro cameras were attached to metal hooks and hung on branches with an extendable 6 m pole 1–5 m from the nest. Each nest was recorded during either building, incubation and/or feeding once (sample size in Table 1). The average GoPro recording duration (min) per nest was 33 ± 3 (mean ± Standard Deviation). We did two to three recordings per day, per nest. We used the first and last recording of each nest for our analyses (Mean ± SD = 1.95 ± 1.1 recordings per nest were used). All recordings were made between 0600 and 1000 during the month of February, which is generally the month with the onset of nest building in Darwin’s finches on Floreana Island.

Solomon coder (Péter 2019) was used to systematically extract information from video recordings to calculate the number of singing events in the neighborhood of the nest. All songs heard were recorded and sampled at a radius of ~ 25 m per nest, as this was the detectability of sound recordings on the GoPro.

### Species identification from song recordings

There are a total of six songbird species in the highlands of Floreana Island, and birds from six other avian taxa (Kleindorfer et al. 2019b) (see Table 2). Songs and calls were compared against a long-term data base managed by Kleindorfer for two decades with 7000 + songs and calls from most species; if a sound could not be identified, the clip was posted on the Galapagos Land Bird WhatsApp group and long-term Galapagos ornithologists (e.g., Birgit Fessl, Thalia Grant, Tui de Roy) provided their expert opinion, which always achieved 100% consensus. The sound identification was also facilitated because only 12 avian land bird taxa (Table 2) are present in the highlands of Floreana Island. The calls of the species listed are identifiable species signals and hence, after training on available recordings and with expert advice, it is likely that all vocalizations were correctly classified to species level.

**Table 2:** Landbirds in the Floreana highlands, shown as Passeriformes and other avian taxa, including also the Whimbrel (*Numenius phaeopus*), a wader commonly seen and heard on the island

Songbirds (Passeriformes)	Other avian Taxa
Small ground finch (*Geospiza fuliginosa)* (E)	Galapagos dove (*Zenaida galapagoensis*) (E)
Medium ground finch (*Geospiza fortis*) (E)	Short-eared owl (*Asio flammeus galapagoensis*)** (**E subspecies**)**
Small tree finch (*Camarhynchus parvulus*) (E)	Dark-billed cuckoo (*Coccyzus melacoryphus)* (N)
Medium tree finch (*Camarhynchus pauper)* (E)	Paint-billed crake* (Mustelirallus erythrops)* (N)
Galapagos flycatcher (*Myiarchus magnirostris)* (E)	Smooth-billed ani (*Crotophaga ani*) (I)
Yellow warbler (*Setophaga petechia aureola*) (E subspecies)	Whimbrel *(Numenius phaeopus*) (N)

The IUCN classification is shown per species as endemic (E), native (N) or introduced (I)

### Data analysis

All data analyses were conducted using R v.4.1.0 (R Core Team 2021). We analyzed the following variables: (1) male age (assessed from plumage categories shown in Figs. 1 and 2), (2) number of total singing events per minute (conspecific + heterospecific songs) in the vicinity of active nests, (3) subset: number of heterospecific singing events per minute, (4) subset: number of conspecific singing events per minute, (5) number of neighboring nests in a 35 m radius of the focal nest (we selected this cut-off as it could have overlapped with the 25 m audible recording range of the GoPro recordings), (6) vegetation canopy cover (% cover), (7) ground cover (%), (8) tree height (m), (9) nesting height (m), and (10) breeding status (nest building, incubation, chick feeding). In terms of nesting outcome, we analyzed variables in relation to whether the nest was depredated or not, but only for the nests recorded in 2020 as this information is not available for 2022 (the field work ended before nesting outcome was known).

To test our predictions, we used linear mixed models with the package ‘lme4’ (Bates et al. 2015) and ‘arm’ (Gelman 2011). The distribution of the residuals and the models’ assumptions were tested and assessed visually using the package ‘DHARMa’ (Hartig 2021). For every prediction, we first conducted a general model without the species distinction and a second model where species was considered separately. First, we explored the general pattern for a difference between younger and older males regardless of the species. Next, we tested if there is a difference in this effect between the species.

We used a pseudo-Bayesian framework with non-informative priors using the packages ‘arm’ (Hilbe 2009; Gelman 2011) and ‘lme4’ (Bates et al. 2015). For every linear mixed model (package ‘lme4’), the restricted maximum-likelihood estimation method was applied. In each model, we applied the function ‘sim’ and carried out 10,000 simulations to obtain the posterior distribution of every estimate, the mean value and the 95% credible interval (CrI) (Korner-Nievergelt 2015). CrIs provide information about uncertainty around the estimates. We considered an effect to be statistically meaningful when the 95% CrI did not overlap with zero. A threshold of 5% is equivalent to the significance level in a frequentist framework (i.e. *p*-value of 0.05; Korner-Nievergelt 2015). For depredation, the response variable was binary (0 = no predation event, 1 = nest depredated) and modeled with a binomial distribution using the logit-link function.

#### Male age and heterospecific singing activity

To analyze whether older males build nests in sites with more heterospecific singing activity, we used two linear-mixed-effect models (REML fit). In both, the response variable was the number of heterospecific songs per minute. In the first model, the explanatory variables were the total number of nests within 35 m (proxy for nesting density) and male age. In the second model, the explanatory variables were the total number of nests within 35 m, the male age and the interaction between male age and species. In both models, Nest ID was included as a random factor to account for repeated measures in a same nest and breeding status to account for the variance across different breeding stages.

#### Male age and conspecific singing activity

To analyze the converse of our predicted association between male age and the number of heterospecific neighbors, we tested if younger males have nest sites with more conspecific neighbors and more conspecific singing activity (and hence, likely, more conspecific competition). We used the same approach as above. Namely, two linear-mixed-effect models (REML fit) with the response variable ‘number of conspecific singing events per minute’. In the first model, the explanatory variables were the male age, the total number of nests within 35 m (proxy for nesting density) and their interaction. In the second model, the explanatory variables were the total number of nests within 35 m and the male age in interaction with species. In both models, Nest ID was included as a random factor to account for repeated measures of the same nest and breeding status to account for differences the breeding phase. Here, the residual diagnostics in both models showed slight (but still acceptable) deviation in one assumption (slight deviation in residual vs. predicted quantiles) that could probably be overcome with larger sample sizes. In 2022, the onset of singing activity occurred later in the season and singing activity was lower, likely because rainfall was lower in 2022 than in 2020 (Floreana data: mean rainfall Feb 2022 = 2.3 mm; mean historic rainfall Feb = 104.1 mm; https://www.galapagosvitalsigns.org); also, there were many zero values for conspecific song in 2022 compared with 2020 though heterospecific song activity had few zero values in either year.

#### Effect of male age on nest site vegetation characteristics

Before assessing if vegetation characteristics of nest sites differed between older and younger males, we first performed a spearman correlation test among all the vegetation variables that we measured: canopy cover, ground cover, tree height and nesting height (Figure S1). We used a Spearman correlation because the different variables were not normally distributed and the distribution ‘types’ varied significantly among each other. Ground cover and canopy covered were highly correlated among each other (*rho* = − 0.491, *p* < 0.001), and this was also the case between tree height and nest height (*rho* = 0.783, *p* < 0.001). Because of this and because previous research identified an association between canopy cover and nesting height on nesting success in this system, we used these two variables in the models to test the association between male age and nest site vegetation characteristics.

The degree of association between male age and nest site canopy cover and nesting height was estimated using one linear model per variable. Each model had male age and species as explanatory variables, and their interaction.

#### Effect of number of singing events (general song-activity) and nest site vegetation on predation outcome

We used binary logistic regression with nest predation outcome (0 = not depredated, 1 = depredated) as the binary-dependent variable against total number of songs per minute, nesting height, and nest site canopy cover as predictor variables.

## Results

### Male age and heterospecific singing activity

**Fig. 3:**
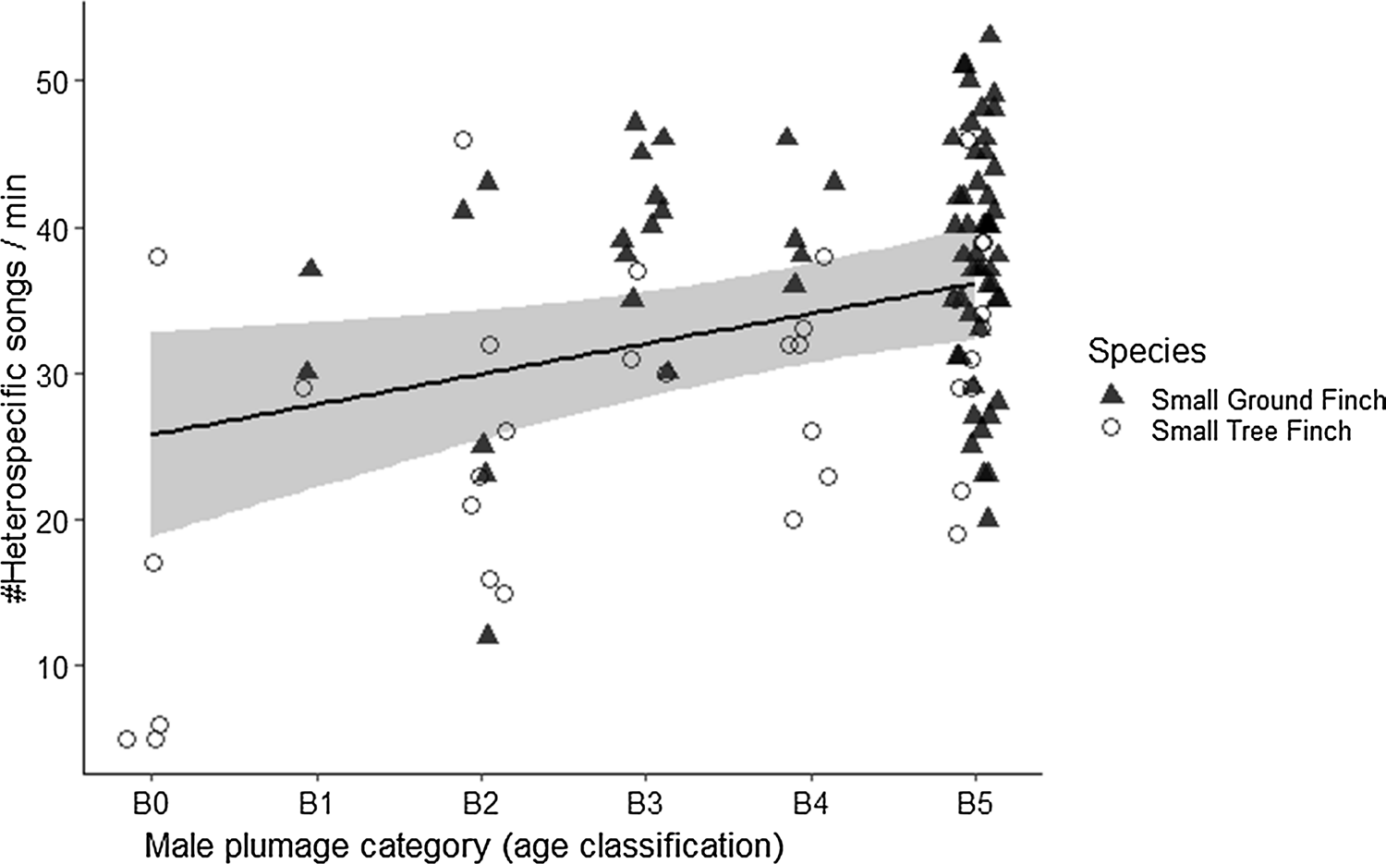
Heterospecific singing activity in relation to male age. X-axis: proportion of black in male plumage category is shown for B0 to B5, which corresponds with age in years from yearling to age 5 + in Darwin’s finches; Y-axis: number of heterospecific singing events per minute. Black line represents the mean estimate, gray ribbon the 95% CrIs and dots the raw data (colour figure online)

Older males had significantly more heterospecific singing activity near the nest (*n* = 55, Mean estimate [95% CrI] = 2.088 [0.447, 3.714], Table S1a) compared to younger males (Fig. 3). This pattern was strongest in Small Ground Finches (Mean effect size [95% CrI] = 2.14 [0.14, 4.19]; *n* = 33), and weak in Small Tree Finches (Mean effect size [95% CrI] = 0.53 [− 2.31, 3.38]; *n* = 22; Table S1). The number of nesting neighbors did not influence the heterospecific singing activity in the territory; neither did the breeding status during which the nesting territories were recorded (Table S1).

### Male age and conspecific singing activity

**Fig. 4:**
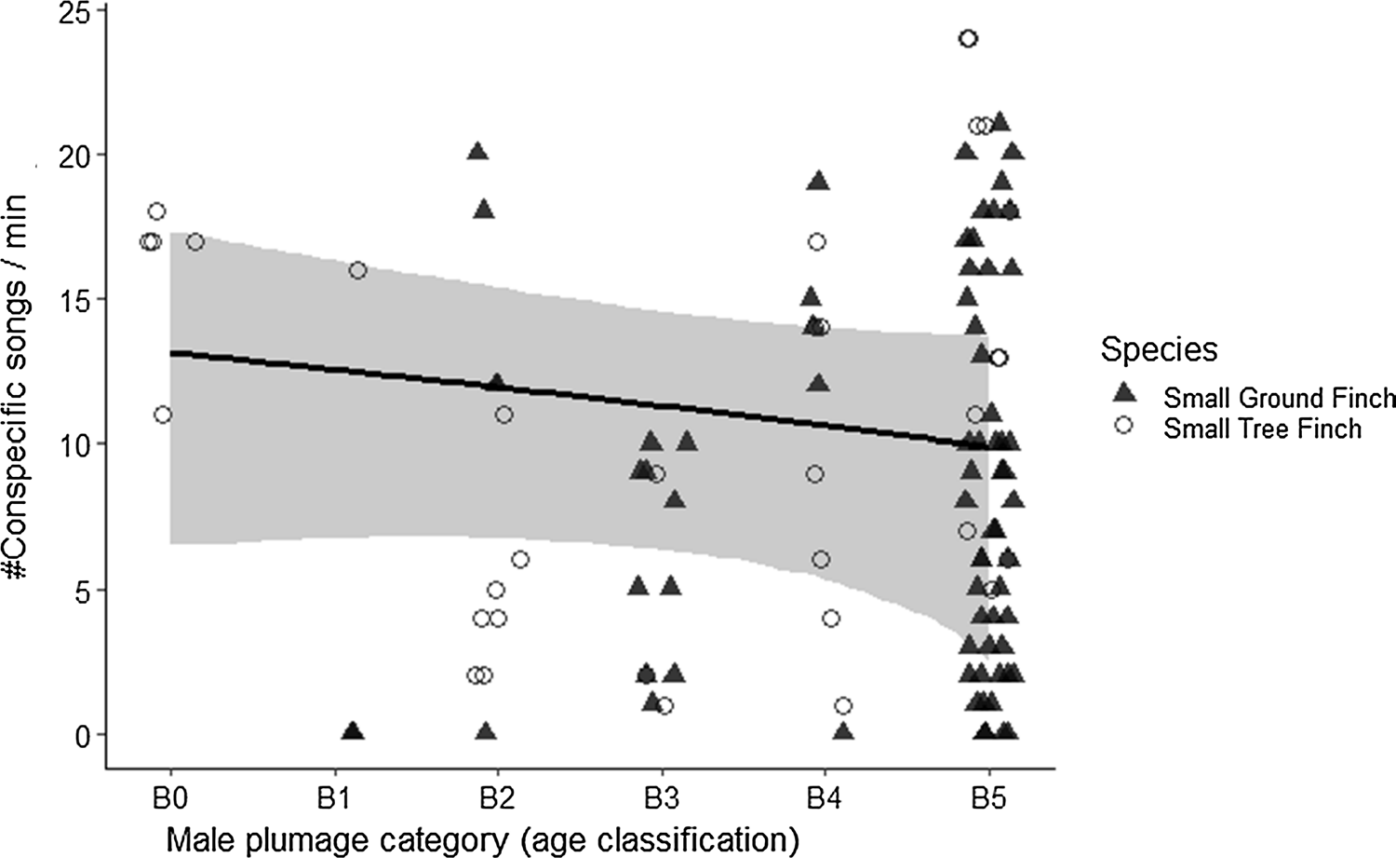
Conspecific singing activity in relation to male age. X-axis: proportion of black in male plumage category is shown for B0 to B5, which corresponds with age in years from yearling to age 5 + in Darwin’s finches; Y-axis: number of conspecific singing events per minute. Black line represents the mean estimate, gray ribbon the 95% CrIs and dots the raw data (colour figure online)

There was no evidence that the level of conspecific singing activity within 25 m radius of a male’s nest changed with male age (Fig. 4). This also held true when accounting for both species separately in the statistical model (Mean effect size [95% CrI] for small ground finch = − 0.35 [− 2.01, 1.31], and for small tree finches = − 0.91 [− 2.54, 0.69]). Rather, the overall number of neighbors was associated with the number of conspecific singing events (*n* = 55, Mean estimate [95% CrI] = 1.738 [0.024, 3.470], Table S2).

### Effect of male age on nest site vegetation characteristics

**Fig. 5:**
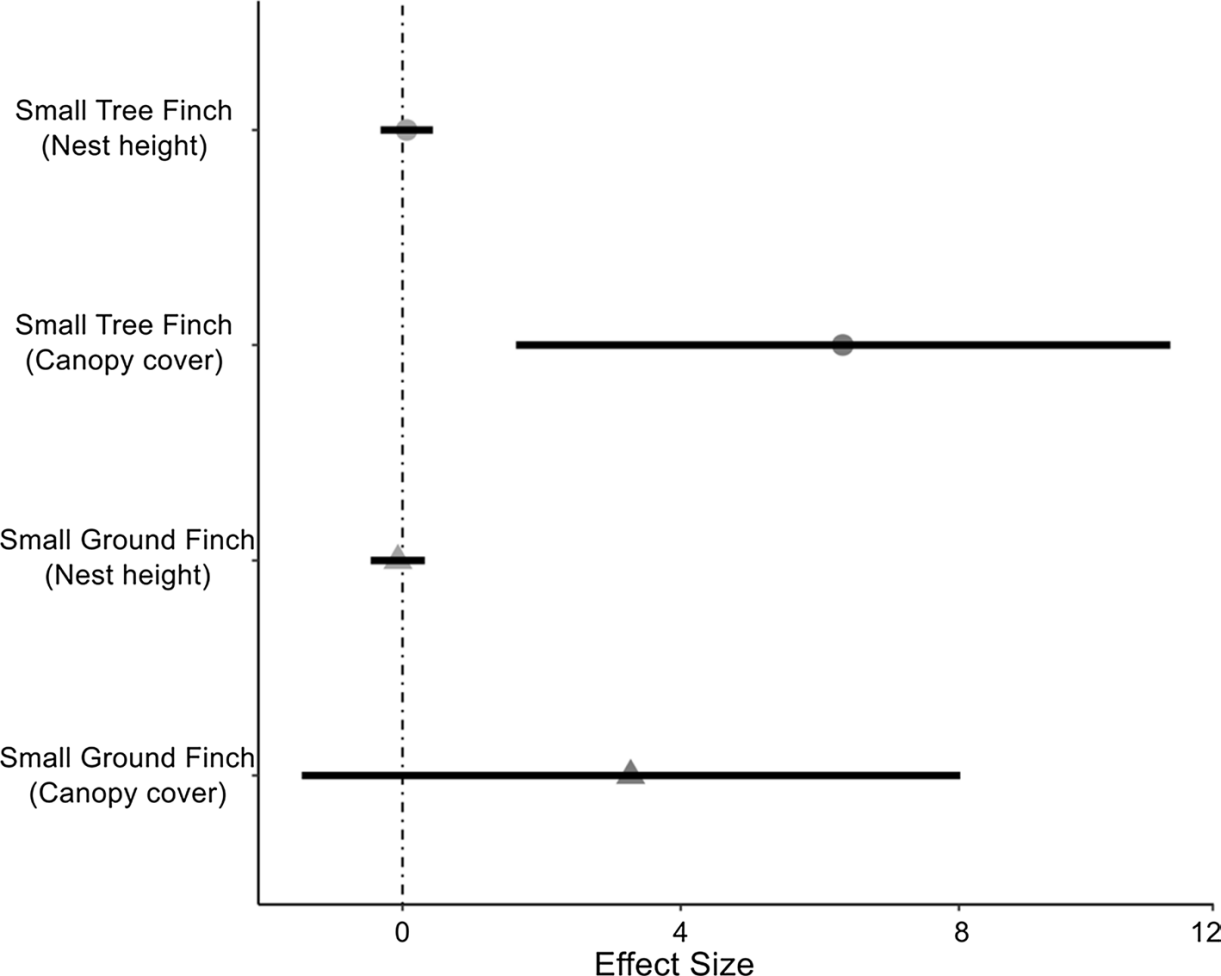
Relationship between age and nest site characteristics. Effect sizes (i.e., slopes; mean and 95% CrI) for the relationship between age and nest height (light blue dots), and between age and canopy cover for small ground (triangles) and small (circles) tree finches. A statistical support larger than 95% (i.e. *p* < 0.05 in a frequentist framework) can be interpreted if a CrI does not overlap zero. Note the large mean effect size of age and canopy cover for small ground finches. Here, the probability of the effect size being larger than zero is 91.2%, equivalent to a frequentist ‘*p’* of 0.09

We tested if nesting height and canopy cover at the nest site was associated with male age. We found the same pattern in both species. The nesting height did not vary in relation to male age (Fig. 5, Mean Slope [95%CrI] for small ground finches = − 0.07 [− 0.45, 0.32], for small tree finches = 0.06 [− 0.32, 0.45], Table S). Regarding vegetation, older male small tree finches nested in areas with significantly more vegetation cover (Fig. 5, Mean slope [95%CrI] = 6.33 [1.72, 11.07], Table S3). Male small ground finches did as well (note the large mean effect size), but with a modest statistical support (Fig. 5, Mean Slope [95%CrI] = 3.32 [− 1.42, 8.11], Table S3).

### Effect of number of singing events (general song-activity) and nest site vegetation on predation outcome

**Fig. 6:**
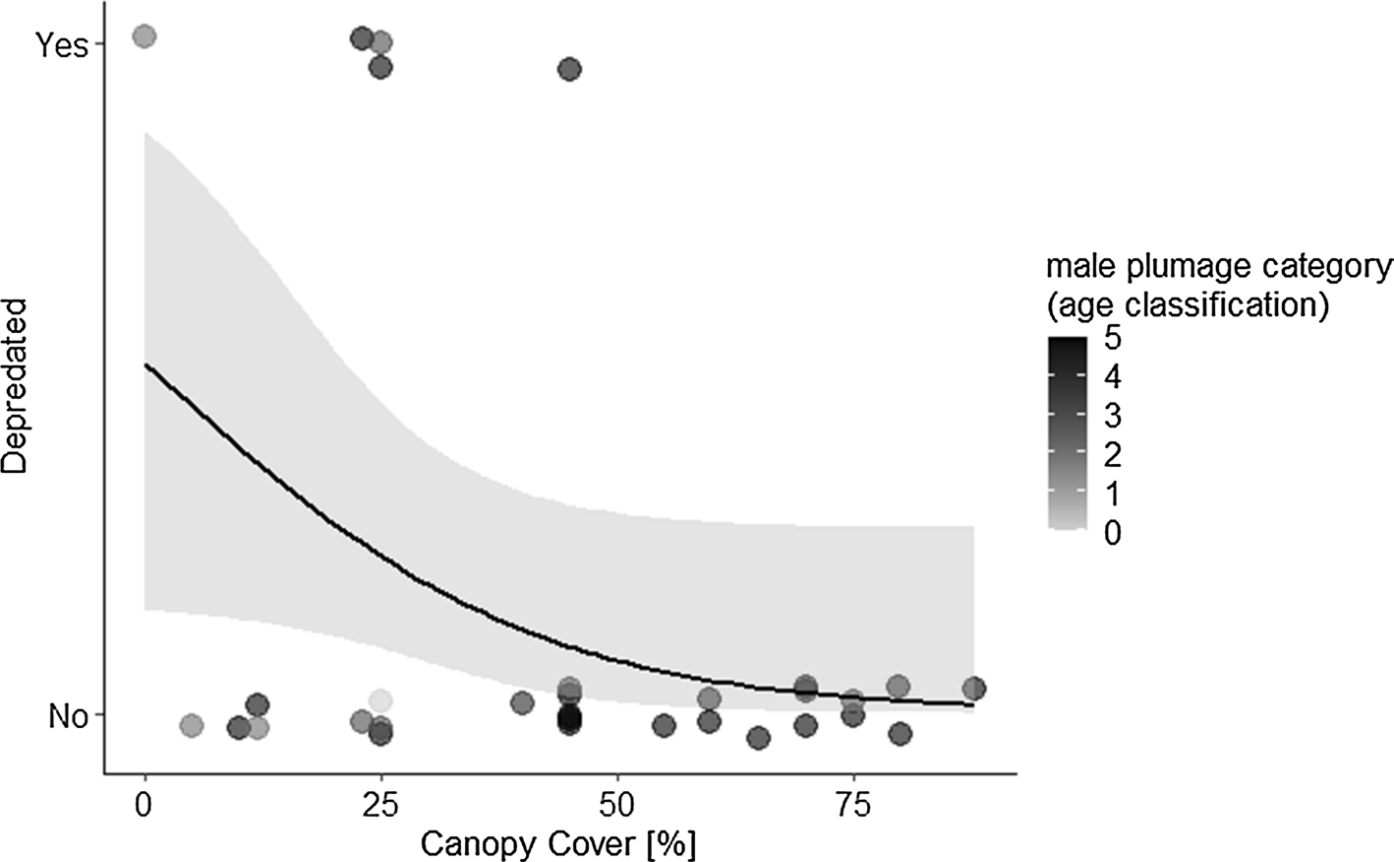
Decreased depredation events with increased canopy cover. The depredation risk decreases with nests that are placed with more canopy cover (*n* = 31, *r* = − 0.38, *p* = 0.050). Black line represents the mean estimate, green ribbon the 95% CrIs and dots the raw data. Color of the dots represents the male age category increasing from sandy brown (B0) to black (B5 +). The percentage of depredated nests was comparable between small tree finches (2/8, 25%) and small ground finches (5/23, 22%) (colour figure online)

We know nesting outcome with certainty for 32 nests (24 Small ground finches and 8 small tree finches). Using binary logistic regression analysis, there was no effect of average number of songs per minute on nest predation (*r* = 0.09, *N* = 32, *p* = 0.847), and no association with nesting height (*r* = 0.528, *p* = 0.324), but more concealed nests had less predation (*r* = − 0.38, *p* = 0.036, Fig. 6) and, specifically, older males had less predation (*r* = − 0.18, *p* = 0.047). The percentage of depredated nests was comparable between small tree finches (2/8, 25%) and Small Ground Finches (5/24, 21%).

## Discussion

The main aim of this study was to test if nest site characteristics, such as vegetation cover and the acoustic neighborhood, differed across male age in two Darwin’s finches: the small tree finch and the small ground finch. As predicted, older males built nests in areas with more vegetation concealment and these nests had less predation. Neither song activity near the nest or nesting height predicted nest predation. A novel finding of this study is that nest sites of older males were exposed to more heterospecific singing activity, and hence such nest sites can be described as occurring in a richer acoustic neighborhood.

The nest sites of younger and older males differed in several ways, and more research is needed to examine the mechanisms for these patterns. Younger males nested in areas with more conspecific neighbors, and older males nested in areas with more heterospecific neighbors, with more vegetation cover surrounding the nest. Perhaps older males outcompete younger males for access to preferred habitat. In support of this idea, we have observed male take-overs of nests, and in all cases, older (B5) males supplanted and usurped younger (B0, B1) males from nests they had built (Kleindorfer et al. 2021b). Because older males also have larger badge size (the extent of black plumage on the crown and chin), it is possible that badge size (rather than age per se) predicts the outcome of agonistic interactions, as has been shown in other systems (Olsson 1994). While younger male Darwin’s finches may occasionally build a nest in an area with dense vegetation cover that also has many heterospecific neighbors, these nests could subsequently be usurped by older males. Younger males may have a preference for the same nest sites as older males but cannot exercise their choice as they are outcompeted by older males. It remains to be tested if younger males actively avoid areas with older males to reduce the probability of nest usurpation and/or paternity loss through cuckoldry.

Our finding that vegetation cover was associated with lower predation risk adds to a body of evidence linking reduced visual conspicuousness of nests with reduced nest predation (Martin and Roper 1988; Colombelli-Négrel and Kleindorfer 2009). On Floreana Island, there are five nest predators of Darwin’s finch nesting contents: introduced Rat (*Rattus rattus*), introduced House Mouse (*Mus musculus*), introduced Cat (*Felis catus*), introduced Smooth-billed Ani (*Crotophaga ani*), and endemic Short-eared Owl (*Asio flammeus galapagoensis*). The number of rats and owls has increased across the past decade (Kleindorfer, unpublished data), not least because owls feed on the ever-increasing rat population. Rats are olfactory hunters that are more common predators at nests closer to the ground and owls are visual hunters that are more common predators at nests higher in the canopy (Kleindorfer et al. 2021b). In a previous study, we showed that nests at intermediate heights sustained the most larvae from the introduced Avian Vampire Fly (Kleindorfer et al. 2016; 2021b), which is the biggest risk factor for the survival of Darwin’s finches (Kleindorfer and Dudaniec 2016; Fessl et al. 2018; McNew and Clayton 2018). Therefore, it is perhaps not surprising that we did not find an effect of nesting height on predation outcome in this study. Future research should explore effects of male age on nesting success and number of vampire flies after the planned predator eradication and predator translocation on Floreana Island managed by the Galápagos National Park Directorate (GNPD). In regard to vegetation cover and biodiversity, our study builds on previous research that found greater biodiversity in areas with greater vegetation diversity (Lantz et al. 2011; Weisshaupt et al. 2011; La Sorte et al. 2020; Geladi et al. 2021), and more bird species in areas with more canopy cover (kirk and Hobson 2001) or vegetation coverage (La Sorte et al. 2020). Our study is also in accordance with previous studies on Santa Cruz island that measured less predation at more concealed nests built by older Darwin’s finch males (Kleindorfer 2007a; Wappl et al. 2020; Heyer et al. 2021).

We acknowledge this is an observational study that aimed to explore whether the acoustic neighborhood of males differed in relation to their age class. Possibly the most novel implication of this study is the finding that offspring of older males were exposed to a richer acoustic neighborhood than offspring of younger males. How such an acoustic neighborhood with more heterospecific singing birds might influence neural development (Rivera et al. 2019; Schroeder and Remage-Healey 2021), gene expression (Antonson et al. 2021), tutor preference (Williams 1990), attention (Soha and Marler 2000; Chen et al. 2016), social learning strategy (Farine et al. 2015) or other vocal production learning pathways (Katsis et al. 2018, 2021; Mariette et al. 2021) remains to be explored. Darwin’s finches are capable of species recognition of song (Ratcliffe and Grant 1995), with reduced response to experimental broadcast of local song versus heterospecific song or foreign dialects (Colombelli-Négrel and Kleindorfer 2021). Perhaps early-life exposure to different song types influences the magnitude of song discrimination, or the efficacy of song transmission from father to son, which remains to be tested.

It is possible that younger males return to natal sites, or sites that look and sound like their natal site, based on vegetation and acoustic cues. Similar processes have been described for habitat imprinting, for example in cuckoos (Teuschl et al. 1998). In a review of the phenomenon of natal habitat preference induction (NHPI), Davis and Stamps (2004) found evidence for NHPI across a broad range of animal taxa. Our study provides a complementary perspective by raising the possibility that acoustic habitat imprinting may play a role in systems with early-life vocal production learning. The findings raise new research questions about mechanisms of nest site selection using acoustic cues, and ontogenetic consequences of different sound exposure for development and sound preference. In the Darwin’s finch system, older males build display nests in areas with more vegetation cover, males compete for access to these nest sites, females select these nests and males, and offspring are—likely as a by-product—exposed to a richer heterospecific neighborhood. A rich acoustic neighborhood, even if it is ‘only’ a by-product of other preferences shaping nest site selection, could have significant impact on offspring development, which future research could explore.

In summary, there is some evidence presented here that older Darwin’s finches of the Galapagos Islands build nests in areas that may be considered local biodiversity hotspots, because they have more vegetation cover and more heterospecific singing neighbors. While the larger badge size of older males could predict occupation of such (potentially) preferred habitats, little research has been done into the possible effects of natal acoustic neighborhood on individual learning strategy, vocal phenotype, or fitness of offspring growing up in those nests. During this Anthropocene era (Lewis and Maslin 2015), when both human activity and infrastructure, and noise and light pollution, are increasingly impacting wildlife, this study provides an example of baseline variance in nest site characteristics in areas without a large human sound footprint. With the observations presented in this study, we hope to spark research interest into consequences of early-life acoustic exposure for development and fitness in vocal production learning species.

### Supplementary Information

Below is the link to the electronic supplementary material.

Supplementary file1 (DOCX 56 kb)

## Data Availability

Data are available on the Flinders University data repository at DOI: 10.25451/flinders.23664561.
