# Supplementary material for: Age effects in Darwin’s finches: older males build more concealed nests in areas with more heterospecific singing neighbors
Source: J Ornithol. 2023 Jul 13;165(1):179–91. doi: 10.1007/s10336-023-02093-5 (PMC10787676; doi:10.1007/s10336-023-02093-5)
Supplement: Supplementary file 1 — Supplementary file1 (DOCX 56 kb) [file 10336_2023_2093_MOESM1_ESM.docx]

# **Supplementary Material**

# **Age effects in Darwin’s finches: older males build more concealed nests in areas with more heterospecific singing neighbours**

Antonia C. Huge^1,2^, Nicolas M. Adreani^1,2^, Diane Colombelli-Négrel^3^, Çağlar Akçay^4,5^, Lauren K. Common^1,2, 3^, Sonia Kleindorfer^1,2,3^

^1^ Konrad Lorenz Research Center for Behavior and Cognition, University of Vienna, Grünau im Almtal, Austria

^2^ Department of Behavioral and Cognitive Biology, University of Vienna, Vienna, 1030, Austria

^3^ College of Science and Engineering, Flinders University, Adelaide, 5001, Australia

^4^ Department of Psychology, Koç University, Istanbul, Turkey

^5^ School of Life Sciences, Anglia Ruskin University, Cambridge, UK

| Table S1a: Relation between male age (plumage coloration) to heterospecific singing activity | |
| --- | --- |
| Heterospecific songs per minute | |
| *Fixed effects (ß) (95 % CrI)* | |
| Male age | **2.088 (0.447, 3.714)** |
| Number of neighbouring nests | -0.341 (-2.645, 1.954) |
| *Random effects (σ^2^) (95 % Crl)* | |
| Nest ID | 4.085 (0.184, 16.478) |
| Breeding status | 29.447 (20.138, 42.170) |

CrI: credible interval. Statistically meaningful effects are marked in bold. Model (linear mixed-effect model, Gaussian error distribution): Heterospecific songs ~ Age + #Nests +

(1 | NestID) + (1 |BreedingStatus)

| Table S1b: Relation between male age (plumage coloration) to heterospecific singing activity including species interaction | |
| --- | --- |
| Heterospecific songs per minute | |
| *Fixed effects (ß) (95 % CrI)* | |
| Male age | 1.011 (-0.978, 2.986) |
| Number of neighbouring nests | -0.308 (-2.395, 1.718) |
| Species (STF against SGF) | **-13.016 (-24.285, -2.050)** |
| Age * Species | 1.404 (-1.297, 4.166) |
| *Random effects (σ^2^) (95 % Crl)* | |
| Nest ID | 0.663 (0.023, 3.271) |
| Breeding status | 17.134 (11.289, 25.445) |

CrI: credible interval. Statistically meaningful effects are marked in bold. Model (linear mixed-effect model, Gaussian error distribution): Heterospecific songs ~ Age * Species + #Nests + (1 | NestID) + (1 |BreedingStatus)

| Table S2: Relation between male age (plumage coloration) to conspecific singing activity including species interaction. | |
| --- | --- |
| Conspecific songs per minute | |
| *Fixed effects (ß) (95 % CrI)* | |
| Male age | -0.394 (-2.049, 1.310) |
| Number of neighbouring nests | **1.738 (0.024, 3.470)** |
| Species (STF against SGF) | 5.159 (-3.181, 13.723) |
| Age * Species | -0.517 (-2.634, 1.574) |
| *Random effects (σ^2^) (95 % Crl)* | |
| Nest ID | 24.527 (18.993, 32.070) |

CrI: credible interval. Statistically meaningful effects are marked in bold. Model (linear mixed-effect model, Gaussian error distribution): Conspecific songs ~ Age * Species + #Nests + (1| NestID)

**Table S3**. Model estimates for the relation between male age and nest site characteristics (Nest height and Canopy cover) in Small Ground Finches (SGF) and Small Tree Finches (STF). Models (linear model, Gaussian error distribution): 1) Nest Height ~ Age * Species; 2) Canopy cover ~ Age * Species.

|  | Nest Height | Canopy cover |
| --- | --- | --- |
| *Fixed effects (ß) (95 % CrI)* | | |
| Intercept (SGF) | 4.24 (2.57, 5.92) | 21.27 (0.67, 41.78) |
| Age | -0.07 (-0.45, 0.31) | 3.31 (-1.43, 8.11) |
| STF | 0.56 (-1.57, 2.71) | -2.39 (-28.57, 24.2) |
| Age * Species (STF) | 0.13 (-0.41, 0.67) | 3.01 (-3.69, 9.58) |


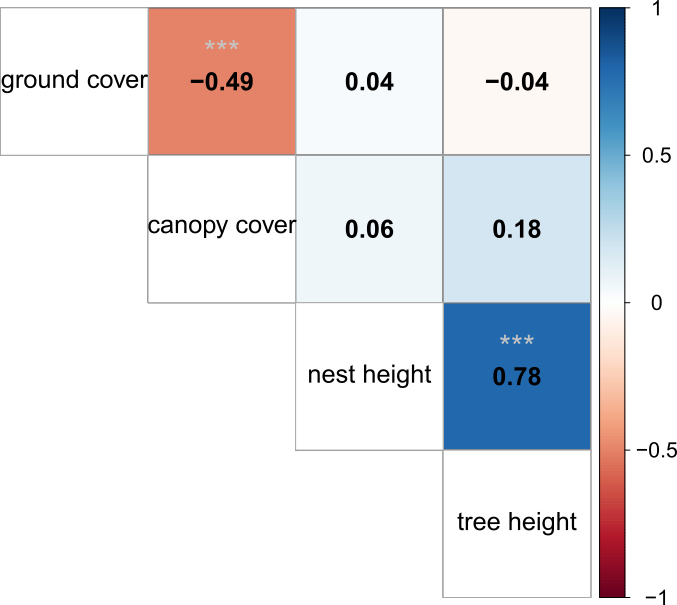


Figure S1: **Correlation matrix of the vegetation characteristics.** Canopy and ground cover are negatively correlated, which is expected. Under conditions of high canopy cover, less sunlight reaches the ground, and vegetation is less likely to grow to great heights in shady environments. Tree height is strongly correlated with nesting height in the tree (both in meters).
